# Supplementary material for: Draft genome of multiple resistance donor plant Sinapis alba: An insight into SSRs, annotations and phylogenetics
Source: PLoS One. 2020 Apr 9;15(4):e0231002. doi: 10.1371/journal.pone.0231002 (PMC7145005; doi:10.1371/journal.pone.0231002)
Supplement: S4 Table — (PDF) [file pone.0231002.s004.pdf]

Repeat Masking Results

Name: Salba.fa

Genomic Sequences

- Input file: Salba.fa
- Number of sequences: 403423
- Total length: 459115215 bp (459115162 bp excl N/X-runs)
- GC level: 36.03 %
- Bases masked: 10963944 bp (2.39 %)

Results Overview

Repeat class/family	Number of elements	Length occupied	Percentage of sequence
**SINEs**	**0**	**0**	**0.00**
Penelope	0	0	0.00
**LINEs**	**0**	**0**	**0.00**
CRE/SLACS	0	0	0.00
L2/CR1/Rex	0	0	0.00
R1/LOA/Jockey	0	0	0.00
R2/R4/NeSL	0	0	0.00
RTE/Bov-B	0	0	0.00
L1/CIN4	0	0	0.00
**LTR elements**	**0**	**0**	**0.00**
BEL/Pao	0	0	0.00
Ty1/Copia	0	0	0.00
Gypsy/DIRS1	0	0	0.00
Retroviral	0	0	0.00
**DNA transposons**	**0**	**0**	**0.00**
hobo-Activator	0	0	0.00
Tc1-IS630-Pogo	0	0	0.00
En-Spm	0	0	0.00
MuDR-IS905	0	0	0.00
PiggyBac	0	0	0.00
Tourist/Harbinger	0	0	0.00
Other (Mirage, P-element, Transib)	0	0	0.00
**Rolling-circles**	**0**	**0**	**0.00**
**Unclassified**	**0**	**0**	**0.00**
**Small RNA**	**0**	**0**	**0.00**
**Satellites**	**0**	**0**	**0.00**
**Simple repeats**	**172132**	**7879629**	**1.72**
**Low complexity**	**52562**	**3091781**	**0.67**

Note: Most repeats fragmented by insertions or deletions have been counted as one element.

Analysis Parameters

Parameter	Value
Search Engine	HMMER
Species	3728 Sinapis alba
Output FASTA	C:\Users\kaush\Desktop\Salba_downstream_analysis\masked_sequences.fasta
Masking Options	Replace by N
Type of repeat	Interspersed repeats, Simple repeats and low complexity DNA

References

- RepeatMasker Open-4.0. Smit, AFA., Hubley, R. and Green, P. . repeatmasker.org/.
- OmicsBox - Bioinformatics made easy. BioBam Bioinformatics. March 3, 2019. www.biobam.com/omicsbox.
