# Supplementary material for: Draft genome of multiple resistance donor plant Sinapis alba: An insight into SSRs, annotations and phylogenetics
Source: PLoS One. 2020 Apr 9;15(4):e0231002. doi: 10.1371/journal.pone.0231002 (PMC7145005; doi:10.1371/journal.pone.0231002)
Supplement: S5 Table — (DOC) [file pone.0231002.s005.doc]

**Supplementary Table S5:**  **KEGG pathway classifications of *S. alba* predicted genes.**

| **S. No.** | **KEGG pathways** | **Number of sequences** | **Number of enzymes** | **Pathway ID** |
| --- | --- | --- | --- | --- |
|  | **1.Metabolism** | | | |
|  | **1.1 Carbohydrate metabolism** | | | |
| 1 | Glycolysis / Gluconeogenesis | 215 | 29 | map00010 |
| 2 | Citrate cycle (TCA cycle) | 22 | 17 | map00020 |
| 3 | Pentose phosphate pathway | 24 | 19 | map00030 |
| 4 | Pentose and glucuronate interconversions | 166 | 17 | map00040 |
| 5 | Mannose type O-glycan biosynthesis | 2 | 2 | map00051 |
| 6 | Galactose metabolism | 92 | 19 | map00052 |
| 7 | Fructose and mannose metabolism | 29 | 24 | map00051 |
| 8 | Ascorbate and aldarate metabolism | 161 | 15 | map00053 |
| 9 | Starch and sucrose metabolism | 431 | 34 | map00500 |
| 10 | Amino sugar and nucleotide sugar metabolism | 52 | 44 | map00520 |
| 11 | Pyruvate metabolism | 128 | 28 | map00620 |
| 12 | Glyoxylate and dicarboxylate metabolism | 36 | 27 | map00630 |
| 13 | Propanoate metabolism | 27 | 15 | map00640 |
| 14 | Butanoate metabolism | 22 | 19 | map00650 |
| 15 | C5-Branched dibasic acid metabolism | 5 | 4 | map00660 |
| 16 | Inositol phosphate metabolism | 55 | 23 | map00562 |
|  | **1.2 Energy metabolism** | | | |
| 17 | Photosynthesis | 2 | 1 | map00195 |
| 18 | Carbon fixation in photosynthetic organisms | 64 | 22 | map00710 |
| 19 | Oxidative phosphorylation | 51 | 6 | map00190 |
| 20 | Carbon fixation pathways in prokaryotes | 23 | 19 | map00720 |
| 21 | Methane metabolism | 32 | 17 | map00680 |
| 22 | Nitrogen metabolism | 15 | 18 | map00910 |
| 23 | Sulfur metabolism | 23 | 15 | map00920 |
|  | **1.3 Lipid metabolism** | | | |
| 24 | Fatty acid biosynthesis | 108 | 13 | map00061 |
| 25 | Fatty acid elongation | 54 | 9 | map00062 |
| 26 | Fatty acid degradation | 231 | 14 | map00071 |
| 27 | Synthesis and degradation of ketone bodies | 6 | 4 | map00072 |
| 28 | Cutin, suberine and wax biosynthesis | 10 | 7 | map00073 |
| 29 | Steroid biosynthesis | 12 | 10 | map00100 |
| 30 | Primary bile acid biosynthesis | 1 | 1 | map00120 |
| 31 | Steroid hormone biosynthesis | 61 | 7 | map00140 |
| 32 | Glycerolipid metabolism | 186 | 23 | map00561 |
| 33 | Glycerophospholipid metabolism | 43 | 33 | map00564 |
| 34 | Ether lipid metabolism | 11 | 8 | map00565 |
| 35 | Sphingolipid metabolism | 90 | 14 | map00600 |
| 36 | Arachidonic acid metabolism | 7 | 5 | map00590 |
| 37 | Linoleic acid metabolism | 5 | 3 | map00591 |
| 38 | alpha-Linolenic acid metabolism | 113 | 12 | map00592 |
| 39 | Biosynthesis of unsaturated fatty acids | 55 | 8 | map01040 |
|  | **1.4 Nucleotide metabolism** |  |  |  |
| 40 | Purine metabolism | 2440 | 52 | map00230 |
| 41 | Pyrimidine metabolism | 34 | 29 | map00240 |
|  | **1.5 Amino acid metabolism** |  |  |  |
| 42 | Alanine, aspartate and glutamate metabolism | 64 | 29 | map00250 |
| 43 | Glycine, serine and threonine metabolism | 136 | 31 | map00260 |
| 44 | Cysteine and methionine metabolism | 56 | 47 | map00270 |
| 45 | Valine, leucine and isoleucine biosynthesis | 11 | 9 | map00290 |
| 46 | Nitrotoluene degradation | 155 | 1 | map00633 |
| 47 | Valine, leucine and isoleucine degradation | 141 | 19 | map00280 |
| 48 | Lysine biosynthesis | 14 | 9 | map00300 |
| 49 | Arginine biosynthesis | 55 | 21 | map00220 |
| 50 | Lysine degradation | 188 | 11 | map00310 |
| 51 | Arginine and proline metabolism | 116 | 22 | map00330 |
| 52 | Histidine metabolism | 101 | 11 | map00340 |
| 53 | Tyrosine metabolism | 153 | 17 | map00350 |
| 54 | Phenylalanine metabolism | 24 | 17 | map00360 |
| 55 | Tryptophan metabolism | 226 | 27 | map00380 |
| 56 | Phenylalanine, tyrosine and tryptophan biosynthesis | 32 | 27 | map00400 |
|  | **1.6 Metabolism of other amino acids** |  |  |  |
| 57 | beta-Alanine metabolism | 112 | 17 | map00410 |
| 58 | Taurine and hypotaurine metabolism | 5 | 4 | map00430 |
| 59 | Phosphonate and phosphinate metabolism | 5 | 5 | map00440 |
| 60 | D-Glutamine and D-glutamate metabolism | 3 | 3 | map00471 |
| 61 | Selenocompound metabolism | 16 | 11 | map00450 |
| 62 | Cyanoamino acid metabolism | 260 | 12 | map00460 |
| 63 | D-Arginine and D-ornithine metabolism | 2 | 2 | map00472 |
| 64 | D-Alanine metabolism | 3 | 2 | map00473 |
| 65 | Glutathione metabolism | 118 | 20 | map00480 |
|  | **1.7 Glycan biosynthesis and metabolism** |  |  |  |
| 66 | N-glycan biosynthesis | 24 | 18 | map00510 |
| 67 | Various types of N-glycan biosynthesis | 17 | 13 | map00513 |
| 68 | Other types of O-glycan biosynthesis | 1 | 1 | map00514 |
| 69 | Glycosaminoglycan biosynthesis - chondroitin sulfate / dermatan sulphate | 1 | 1 | map00532 |
| 70 | Glycosaminoglycan biosynthesis - heparan sulfate / heparin | 1 | 1 | map00534 |
| 71 | Glycosaminoglycan biosynthesis - keratan sulfate | 1 | 1 | map00533 |
| 72 | Glycosaminoglycan degradation | 72 | 4 | map00531 |
| 73 | Glycosylphosphatidylinositol(GPI)-anchor biosynthesis | 1 | 1 | map00563 |
| 74 | Glycosphingolipid biosynthesis - lacto and neolacto series | 4 | 2 | map00601 |
| 75 | Glycosphingolipid biosynthesis – globo and isoglobo series | 11 | 4 | map00603 |
| 76 | Glycosphingolipid biosynthesis - ganglio series | 70 | 2 | map00604 |
| 77 | Lipopolysaccharide biosynthesis | 8 | 8 | map00540 |
| 78 | Peptidoglycan biosynthesis | 4 | 3 | map00550 |
| 79 | Other glycan degradation | 140 | 8 | map00511 |
|  | **1.8 Metabolism of cofactors and vitamins** |  |  |  |
| 80 | Thiamine metabolism | 2411 | 11 | map00730 |
| 81 | Riboflavin metabolism | 124 | 13 | map00740 |
| 82 | Vitamin B6 metabolism | 51 | 7 | map00750 |
| 83 | Nicotinate and nicotinamide metabolism | 61 | 19 | map00760 |
| 84 | Pantothenate and CoA biosynthesis | 20 | 18 | map00770 |
| 85 | Biotin metabolism | 7 | 9 | map00780 |
| 86 | Lipoic acid metabolism | 2 | 2 | map00785 |
| 87 | Folate biosynthesis | 27 | 18 | map00790 |
| 88 | One carbon pool by folate | 14 | 13 | map00670 |
| 89 | Porphyrin and chlorophyll metabolism | 83 | 28 | map00860 |
| 90 | Ubiquinone and other terpenoid-quinone biosynthesis | 14 | 14 | map00130 |
|  | **1.9 Metabolism of terpenoids and polyketides** |  |  |  |
| 91 | Terpenoid backbone biosynthesis | 59 | 26 | map00900 |
| 92 | Monoterpenoid biosynthesis | 12 | 7 | map00902 |
| 93 | Retinol metabolism | 186 | 4 | map00830 |
| 94 | Sesquiterpenoid and triterpenoid biosynthesis | 5 | 4 | map00909 |
| 95 | Diterpenoid biosynthesis | 4 | 4 | map00904 |
| 96 | Biosynthesis of vancomycin group antibiotics | 1 | 1 | map01055 |
| 97 | Acarbose and validamycin biosynthesis | 1 | 1 | map00525 |
| 98 | Carotenoid biosynthesis | 27 | 6 | map00906 |
| 99 | Zeatin biosynthesis | 14 | 5 | map00908 |
| 100 | Insect hormone biosynthesis | 92 | 2 | map00981 |
| 101 | Limonene and pinene degradation | 93 | 2 | map00903 |
| 102 | Geraniol degradation | 4 | 4 | map00281 |
| 103 | Brassinosteroid biosynthesis | 1 | 1 | map00905 |
| 104 | Biosynthesis of ansamycins | 1 | 1 | map01051 |
| 105 | Polyketide sugar unit biosynthesis | 1 | 3 | map00523 |
| 106 | Biosynthesis of siderophore group nonribosomal peptides | 1 | 1 | map01053 |
|  | **1.10 Biosynthesis of other secondary metabolites** |  |  |  |
| 107 | Phenylpropanoid biosynthesis | 626 | 17 | map00940 |
| 108 | Stilbenoid, diarylheptanoid and gingerol biosynthesis | 4 | 3 | map00945 |
| 109 | Flavonoid biosynthesis | 9 | 8 | map00941 |
| 110 | Flavone and flavonol biosynthesis | 48 | 5 | map00944 |
| 111 | Anthocyanin biosynthesis | 3 | 2 | map00942 |
| 112 | Betalain biosynthesis | 1 | 1 | map00965 |
| 113 | Indole alkaloid biosynthesis | 2 | 1 | map00901 |
| 114 | Monobactam biosynthesis | 7 | 5 | map00261 |
| 115 | Isoquinoline alkaloid biosynthesis | 12 | 8 | map00950 |
| 116 | Carbapenem biosynthesis | 1 | 2 | map00332 |
| 117 | Tropane, piperidine and pyridine alkaloid biosynthesis | 11 | 6 | map00960 |
| 118 | Caffeine metabolism | 158 | 3 | map00232 |
| 119 | Glucosinolate biosynthesis | 5 | 4 | map00966 |
| 120 | Penicillin and cephalosporin biosynthesis | 2 | 1 | map00311 |
| 121 | Biosynthesis of antibiotics | 456 | 182 | map01130 |
| 122 | Streptomycin biosynthesis | 8 | 9 | map00521 |
| 123 | Neomycin, kanamycin and gentamycin biosynthesis | 2 | 2 | map00524 |
| 124 | Novobiocin biosynthesis | 7 | 5 | map00401 |
| 125 | Aflatoxin biosynthesis | 3 | 1 | map00254 |
| 126 | Biosynthesis of secondary metabolites-other antibiotics | 1 | 1 | map00998 |
| 127 | Biosynthesis of secondary metabolites-unclassified | 2 | 2 | map00999 |
| 128 | Benzoxazinoid biosynthesis | 4 | 1 | map00402 |
|  | **1.11 Xenobiotics biodegradation and metabolism** |  |  |  |
| 129 | Benzoate degradation | 6 | 7 | map00362 |
| 130 | Aminobenzoate degradation | 32 | 9 | map00627 |
| 131 | Fluorobenzoate degradation | 1 | 1 | map00364 |
| 132 | Chloroalkane and chloroalkene degradation | 180 | 3 | map00625 |
| 133 | Toluene degradation | 24 | 2 | map00623 |
| 134 | Ethylbenzene degradation | 1 | 1 | map00642 |
| 135 | Styrene degradation | 8 | 6 | map00643 |
| 136 | Polycyclic aromatic hydrocarbon degradation | 1 | 1 | map00624 |
| 137 | Atrazine degradation | 1 | 1 | map00791 |
| 138 | Bisphenol degradation | 1 | 1 | map00363 |
| 139 | Xylene degradation | 22 | 1 | map00622 |
| 140 | Caprolactam degradation | 51 | 5 | map00930 |
| 141 | Naphthalene degradation | 88 | 1 | map00626 |
| 142 | Steroid degradation | 6 | 3 | map00984 |
| 143 | Metabolism of xenobiotics by cytochrome P450 | 219 | 7 | map00980 |
| 144 | Drug metabolism - cytochrome P450 | 265 | 7 | map00982 |
| 145 | Drug metabolism - other enzymes | 1108 | 19 | map00983 |
|  | **2. Genetic Information Processing** |  |  |  |
|  | **Translation** |  |  |  |
| 146 | Aminoacyl-tRNA biosynthesis | 25 | 22 | map00970 |
|  | **3. Environmental Information Processing** |  |  |  |
|  | **Signal transduction** |  |  |  |
| 147 | Phosphatidylinositol signaling system | 86 | 22 | map04070 |
| 148 | mTOR signaling pathway | 2 | 1 | map04150 |
|  | **4. Organismal Systems** |  |  |  |
|  | **Immune system** |  |  |  |
| 149 | T cell receptor signaling pathway | 586 | 2 | map04660 |
| 150 | PD-L1 expression and PD-1 checkpoint pathway in cancer | 585 | 1 | map05235 |
| 151 | Th1 and Th2 cell differentiation | 585 | 1 | map04658 |
|  | **5. Human Diseases** |  |  |  |
|  | **Drug resistance** |  |  |  |
| 152 | beta-Lactam resistance | 2 | 1 | map00312 |
