# Supplementary figures and images for: Draft genome of multiple resistance donor plant Sinapis alba: An insight into SSRs, annotations and phylogenetics
Source: PLoS One. 2020 Apr 9;15(4):e0231002. doi: 10.1371/journal.pone.0231002 (PMC7145005; doi:10.1371/journal.pone.0231002)

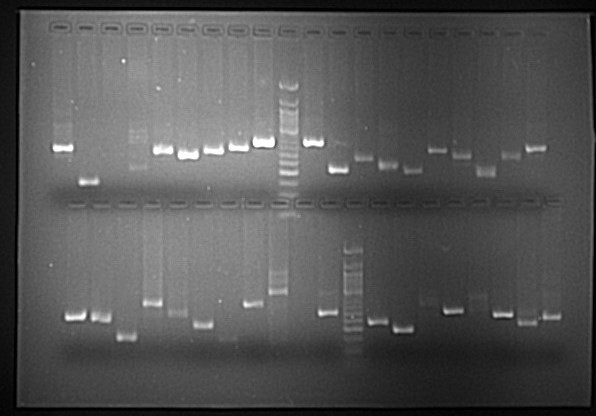

Supplement: S1 Fig — (TIF) [file pone.0231002.s006.tif]
