# Supplementary material for: Draft genome of multiple resistance donor plant Sinapis alba: An insight into SSRs, annotations and phylogenetics
Source: PLoS One. 2020 Apr 9;15(4):e0231002. doi: 10.1371/journal.pone.0231002 (PMC7145005; doi:10.1371/journal.pone.0231002)
Supplement: Attachment — Submitted filename: Rebuttal letter.docx [file pone.0231002.s007.docx]

**Points raise by Academic editor with their answers**:

1. In this work, the authors have used alredy published at 2006 (Zhang X et al., "De novo Transcriptome Analysis of Sinapis alba in Revealing the Glucosinolate and Phytochelatin Pathways", Front Plant Sci, 2016 Mar 4;7:259) publicly available Illumina NGS sequence data of S. alba genome (SRR3490913, SRR3490914, SRR1799170, SRR1799171) for de-novo assembly, gene prediction with annotation and identification of SSR markers.

**Answer**: Academic editor wrote that authors used already published work of Zhang et al. (2016) as they did work on Sinapis alba transcriptome while I have reported my work on GENOME that are two different basic study materials. The academic editor wrote that NGS sequence data is publicly available with accessions SRR3490913, SRR3490914, SRR1799170, SRR1799171. These are raw sequencing data that cannot be used as it is available and also not a part of study of Zhang et al. (2016) while it is my study material and I have processed this raw data (FASTQ to FASTA) and made it in usable form and submitted to NCBI now. Zhang et al. (2016) made their study on transcriptome that are available with accessions [SRR2961888](https://www.ncbi.nlm.nih.gov/nucleotide/SRR2961888), [SRR2961889](https://www.ncbi.nlm.nih.gov/nucleotide/SRR2961889), and [SRR2961890](https://www.ncbi.nlm.nih.gov/nucleotide/SRR2961890). Thus both studies were made on two different genomic materials. Thus his statement that it is repeated study is totally misleading.

2. The main result of the work is genes annotation, SSR detection, primer design and validation.

**Answer**: The main result of the study is development of de-novo assembly, structural and functional annotation of assembly, gene prediction and their annotation, SSRs development, characterization and validation, reconstruction of defence related pathway genes, Retrotransposon abundance,

3. Unfortunately, the virtual detection of potential SSR loci in NGS data is not of practical importance. The authors needed to check all potential SSR pairs of primers on different Sinapis alba varieties or lines and publish only real and useful SSR pairs.

**Answer**: Sinapis alba is well known wild plant of Brassicaceae family having genomic resistance against *Alternaria brassicae*, *Sclerotinia sclerotiorum* pathogens with High temperature, drought, pod shattering, insect tolerance etc. The genome sequence was not publicly available in FASTA format before this study that can be used for molecular breeding and other studies. As genome was not available, genic molecular markers were also not available publicly. The SSRs are very important tool for identification of genomic regions carrying regions of resistance. That’s why this plant was very little studied. Thus, I am completely disagreeing with his statement that detection of SSRs does not have practical importance (Vieira et al 2016. Microsatellite markers: what they mean and why they are so useful. [Genet Mol Biol](https://www.ncbi.nlm.nih.gov/pmc/articles/PMC5004837/). 2016 Jul-Sep; 39(3): 312–328). I have identified >70,000 SSRs that cannot be practically checked by any researcher. However, I have randomly checked 54 SSRs and included the results in my study. Zhang et al. (2016) identified only 14,727 SSRs but not validated any one and they also claim in his study that it is first large collection of *S. alba* SSRs. However, I have identified 5 times more genic SSRs.

4. Authors wrote:
   “For validation of randomly selected 51 SSR markers, the young leaves of field-grown S. alba plants were used for extraction and purification of genomic DNA as reported earlier [20].” However, there is no variety information or specific information.
   I see that part of the SSR primers from supplemental table 3 are themselves part of the microsatellite sequence. Therefore, such primers cannot be used for SSR analysis:

   cactctcactctctctctctctctc
   ttcctctccctctccctctc
   ttctctctctctctctctctcactc
   ggcgcaaagagagagagaaa
   cgtcatctctctctctcgcaat
   accgacaaacaaactcgaca
   gagacggagaaccagacgac
   tgtgtgttggggatagatgg
   taaggaagcaagaggcagga
   tttcgaactccctctctctcc

**Answer**: The Sinapis alba used in this study was Bharatpur strain and used 54 SSRs for validation. I have selected mono allelic SSRs randomly for validation and do not claim that they must be included by others in their studies. They may be from microsatellite sequences or not. Thus statement of academic editor must not have importance in rejection. Now, I have replaced all these primer pairs from my study with other primer pairs.
