# Supplementary material for: Draft genome of multiple resistance donor plant Sinapis alba: An insight into SSRs, annotations and phylogenetics
Source: PLoS One. 2020 Apr 9;15(4):e0231002. doi: 10.1371/journal.pone.0231002 (PMC7145005; doi:10.1371/journal.pone.0231002)
Supplement: Attachment — Submitted filename: reponse to reviewers.docx [file pone.0231002.s008.docx]

**Points raise by Academic editor with their answers**:

1. Did you get the sequences from NCBI portal or did you produce the sequences with the help of JGI as they write in assembly preparation (this part needs to be clear for the readers). I suppose that you produce the sequences but (as also other editor refers) there is no information of how was the DNA generated, leaf or root and the procedures. Also did you perform 4 different experiments or 4 different runs (this is also mentioned from the other editor's review).

**Ans**. We have got raw (FASTQ) whole genome NGS data of *Sinapis alba* from Sequence Read Archive (SRA) of NCBI originally developed and submitted by JGI, USA. We have mentioned this point in material & methods and results & discussion. We also acknowledge Department of Energy, JGI, USA for using his data in this study. The data was generated from 4 different runs and it is mentioned in manuscript.

2. This is not a whole genome research but contig generation (this is also mentioned from the other editor review).

**Ans**. This is whole genome research because we have developed draft assembly from whole genome raw sequences and generated about 83 percent (459 Mb) genome of Sinapis alba in form of contigs. During assembly development, some sequences were removed by softwares.

3. Authors did only validation in one strain of S. alba, and they do not write how many plants of this strain have been used.

**Ans**. The SSRs were generated from draft assembly of Sinapis alba (Strain: S2 GC0560-79) and successfully validated in another strain of Sinapis alba (DRMR 2183). The DNA of single S. alba plant was used for validation of SSRs and this is mentioned in manuscript.

4. You have to specify the ploidy of the crop as also other editor mentioned and furthermore to do a synteny analysis because you found so many SSRs but without a previous genetic map further validation of the SSRs must be performed.

**Ans**. The Sinapis alba is diploid (2n= 24) plant of brassicaceae family which is mentioned in introduction section of manuscript. Now, we are reporting more than 260 validated SSR markers in S. alba (DRMR 2183) in revised manuscript from our earlier validation of 54 markers. This larger number of SSR marker validation should be sufficient for any further studies on S. alba.

5. Furthermore whole Abstract should be rewritten especially in the part of background and in the conclusion section. Also abstract has more than 300 words (PlOs One requires that abstract should not exceed 300 words).

**Ans**. The abstract is revised as per your suggestion and limited within 300 words.

6. My concern is also according to the published article from Zhang X et al., 2006"De novo Transcriptome Analysis of Sinapis alba in Revealing the Glucosinolate and Phytochelatin Pathways", Front Plant Sci, 2016 Mar 4;7:259 authors also produced 14,727 SSRs and there is no discussion about advantages & disadvantages for use of SSR primers produced in current manuscript instead of using SSR primers from Zang et al 2016.I can understand that you used different material but I would expect better explanation about this point.

**Ans**. We have generated 5 times more SSRs from Zhang et al 2016 and discussed the advantages of present study over Zhang’s results in detail. We hope the academic editor will be satisfied with our explanations in revised manuscript.
